# Initial Case Report of Robot‐Assisted Radical Cystectomy With Intracorporeal Neobladder Using hinotori Surgical Robot System

**DOI:** 10.1002/iju5.70016

**Published:** 2025-03-12

**Authors:** Hiromitsu Watanabe, Kyohei Watanabe, Yuto Matsushita, Keita Tamura, Daisuke Motoyama, Atsushi Otsuka, Teruo Inamoto, Hideaki Miyake

**Affiliations:** ^1^ Department of Urology Hamamatsu University School of Medicine Hamamatsu Japan; ^2^ Department of Developed Studies for Advanced Robotic Surgery Hamamatsu University School of Medicine Hamamatsu Japan; ^3^ Department of Urology Kobe University Graduate School of Medicine Kobe Japan

**Keywords:** bladder cancer, hinotori surgical system, robot‐assisted radical cystectomy

## Abstract

**Introduction:**

Robot‐assisted radical cystectomy (RARC) is becoming the standard treatment for bladder cancer patients. While this surgery using da Vinci has been widely reported, this report describes the initial experience of RARC with intracorporeal neobladder using the hinotori surgical robot system.

**Case Presentation:**

The patient was a 73‐year‐old man with muscle‐invasive bladder cancer who underwent neoadjuvant chemotherapy followed by RARC with intracorporeal neobladder using hinotori. Surgery was successfully performed with a total operative time of 430 min, time using the robotic system of 375 min, and no intraoperative complications or need for blood transfusion. Postoperative recovery was favorable, with the patient discharged on day 12, and satisfactory continence was achieved.

**Conclusions:**

This is the initial case report of RARC with intracorporeal neobladder using hinotori, providing a potentially comparable alternative to conventional surgical systems for RARC.

**Summary**

- We reported the initial experience of robot‐assisted radical cystectomy with intracorporeal neobladder using hinotori.
- The operative time was 430 min; there was no blood transfusion, and the patient was discharged on postoperative day 12 without any complications.
- This surgery could be successfully completed and result in a favorable perioperative outcome.

**Abbreviations**
3D: 3‐dimensional
GC: gemcitabine and cisplatin
ICIC: intracorporeal ileal conduit
ICNB: intracorporeal neobladder
LOS: length of stay
MIBC: muscle‐invasive bladder cancer
MIS: minimally invasive surgery
NAC: neoadjuvant chemotherapy
ORC: open radical cystectomy
PLND: pelvic lymph node dissection
POD: postoperative day
PS: performance status
RAA: robot‐assisted adrenalectomy
RAPN: robot‐assisted partial nephrectomy
RARC: robot‐assisted radical cystectomy
RARN: robot‐assisted radical nephrectomy
RARP: robot‐assisted radical prostatectomy
TURBT: transurethral resection of bladder tumor

## 1 Introduction

Over the past two decades, MIS has evolved alongside surgical robotic systems, particularly with the da Vinci surgical system (Intuitive Surgical Inc., Sunnyvale, CA, USA), which has been pivotal in advancing robotic surgery worldwide. In the field of bladder surgery, Menon et al. were the first to document RARC using da Vinci [1]. Ileal conduit remains the most frequently conducted procedure, while the rate of neobladder has markedly increased [2]. However, there have been numerous previous reports detailing RARC conducted with the da Vinci surgical system. Thus, this is the first report of initial experience with RARC featuring ICNB using the hinotori surgical robot system (Medicaroid Corporation, Kobe, Hyogo, Japan).

## 2 Case Presentation

**FIGURE 1:**
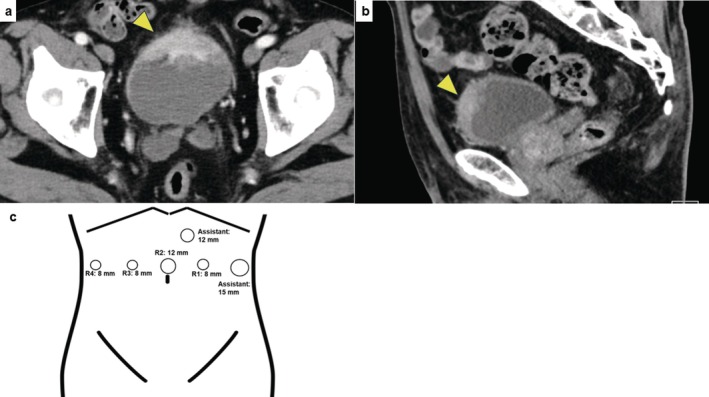
Contrast‐enhanced computed tomography in this case. Tumor located in bladder apex (indicated by arrows) with extraluminal invasion; (a) axial image, (b) sagittal image. (c) Trocar positions for robot‐assisted radical cystectomy with intracorporeal urinary diversion. R1 to R4 and assistant 15 mm were aligned at 8‐cm intervals. AirSeal iFS was used for assistant 12 mm. R1–4: Robot ports 1–4. Monopolar curved scissors, bipolar Maryland forceps, versatile grasping forceps, and a needle holder were used for cystectomy and pelvic lymph node dissection, and universal and Croce grasping forceps were additionally used for urinary diversion.

**FIGURE 2:**
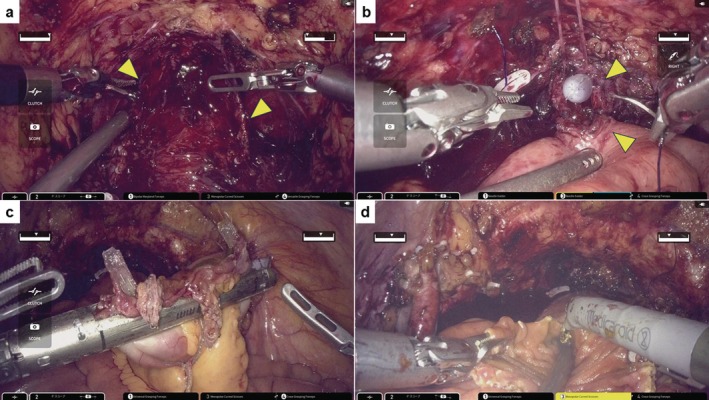
(a) The bladder and prostate were resected, with both neurovascular bundles preserved (indicated by arrows). (b) Urethral‐ileal anastomosis (indicated by arrows) was created with a running suture by 4–0 Stratafix (Ethicon, Johnson & Johnson, Cincinnati, OH, USA). (c) Signia stapling system (Medtronic, Minneapolis, MN, USA) was used for functional end‐to‐end anastomosis. (d) Detubularization of separated ileum.

**FIGURE 3:**
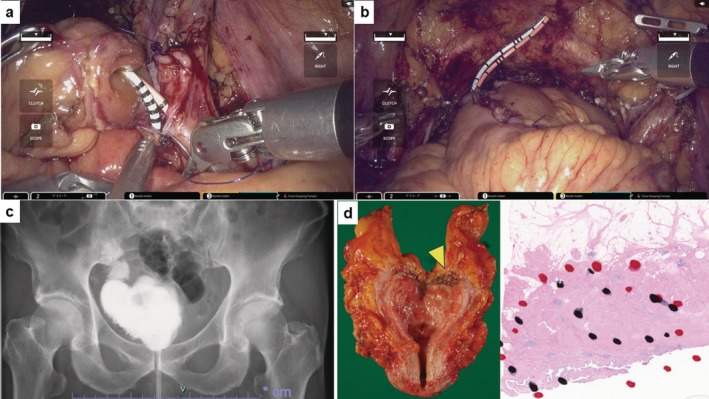
(a) Ureterointestinal anastomosis was performed with the Wallace method. (b) Completed creation of the neobladder. (c) Cystogram of the neobladder created using the Karolinska‐modified Studer method on POD 9. No urine leakage was observed. (d) Macroscopic findings of tumor located in bladder apex (arrows indicated) with extraluminal invasion (left image). Microscopic findings of hematoxylin and eosin staining showing urothelial carcinoma, pT3a, and the surgical margin was negative (right image).

A 73‐year‐old man (PS: 0) who had hypertension and diabetes mellitus was referred to our institute with MIBC; cT3N0M0 (Figure 1a,b). Baseline characteristics are listed in Table 1a. Initially, he received NAC; the best response was PR based on the RECIST guideline (version 1.1). After the chemotherapy, RARC and ICNB using hinotori were performed. This surgery was completely conducted by a single surgeon (H.W.). The surgeon has performed over 30 RARC surgeries using da Vinci and 40 using hinotori. At our institute, we have experienced ICNB using hinotori but never using da Vinci. The present protocol was approved by the institution's optimally constituted ethics committee (Approval No. 21–090). These procedures were carried out as follows. In brief, a transperitoneal approach was employed, with four trocars for the robotic arms and two for the assistant's ports, including AirSeal iFS (CONMED Japan KK, Tokyo, Japan) (Figure 1c). The LigaSure sealing system and Signia stapling system (Medtronic, Minneapolis, MN, USA) were used to seal the bladder pedicle and for bowel reconstruction. The patient was placed under general and epidural anesthesia and in a 20° head‐down position. First, radical cystectomy followed by bilateral nerve‐sparing and PLND was performed. Bilateral ureters were dissected until the ureterovesical junction. The ureters were clipped and cut, and a rapid histological examination confirmed no malignancy. Opening Denonvilliers' fascia up to the level of the urethra was followed by opening the endopelvic fascia, and then dissecting and cutting the bladder pedicles but sparing the neurovascular bundle. Anterior dissection of the bladder was performed up to the prostatic apex, and the dorsal vein complex and urethra were cut (Figure 2a). The bladder was completely resected. After PLND, the neobladder was created. The Karolinska‐modified Studer method was applied. After selecting a 40‐cm intestinal segment, the posterior wall was reinforced with a running suture between the median fibrous raphe and the intestinal wall, the so‐called “Rocco stitch.” Next, a urethral‐ileal anastomosis was created, followed by the segment being separated by a stapler (Figure 2b). Iliac reconstruction was performed by functional end‐to‐end anastomosis (Figure 2c). All areas except for an oral 10‐cm portion of the ileum were detubularized (Figure 2d). This 10‐cm portion was used for Wallace ureterointestinal anastomosis in the afferent limb (Figure 3a). The detubularized ileum was running‐sutured and closed to create a neobladder (Figure 3b). All procedures were successfully performed, and good clinical outcomes were achieved (Table 1b, Figure 1c). The pathological status of the excised tumor was urothelial carcinoma pT3a, with no lymph node metastasis, and the surgical margin was negative (Figure 3d).

**TABLE 1:** Patient data of baseline from postoperative outcomes and features of two different surgical robotic systems.

(a) Baseline characteristics	
Charlson comorbidity index	8
American Society of Anesthesiologists score	1
BMI, kg/m^2^	21.9
Hemoglobin, g/dL	12.2
Histological type, cT stage	Urothelial carcinoma, cT3b
Neoadjuvant chemotherapy, number of courses	Gemcitabine/Cisplatin, 3

(b) Peri‐ and postoperative outcomes	
Operative time, min	430
Time using robotic system, min	375
Bowel reconstruction and urinary diversion time, min	215
Pelvic lymph node dissection	Extended
No. of lymph nodes dissected	49
Estimate blood loss, mL	425
Blood transfusion	0
Intraoperative complications	0
Mechanical device errors	0
Adherence to ERAS protocol	Yes
Time to mobilization, POD	1
Time to solid intake, POD	4
Time to flatus, POD	7
Time to removal of ureteral stents, POD	8
Time to removal of urethral catheter, POD	10
Length of hospital stay after surgery, POD	12
Surgical margin	Negative
Perioperative (hospitalized) complications	0
30–180‐day complications and readmissions	0

(c) Voiding outcome based on diary (POD 180)	
FDV, mL	228
NPI, %	39.3
Mean daytime pads used, *n* (pad wetness, g)	1 (0)
Mean nighttime pads used, *n* (pad wetness, g)	1 (0)
Clean intermittent catheterization	No

(d) Subjective and objective differences between the two surgical robot systems		
	hinotori	da Vinci
Motion/feeling	Straightforward and human‐like manner	Sophisticated and sharper
Vision	Full high vision	4K
Arms	4 arms, and docking the port to arm.	4 arms, and free‐docking (computer calibrated)
Device	Original sealing and stapling instrument	Sealing and stapling are conducted by assistant surgeon
Cost (RARC surgical device)	$3279	$3617

Satisfactory postoperative continence was achieved. According to the voiding diary of POD180, the patient was wearing preventive pad (safty‐pad) both during the day and at night. (Table 1c). In addition, no marked complications or readmissions occurred (Table 1b).

## 3 Discussion

In recent years, various novel robotic surgical systems have been required and used in real‐world clinical settings [3]. Among these platforms, hinotori is the first surgical system from Japan [4]. Table 1d outlines the differences between hinotori and da Vinci. hinotori has several iconic and advantageous characteristics. First, each operating arm, with its eight axes, facilitates “human‐hand‐like movement.” Second, the trocar position is calibrated by computer software, and so each arm can be used without docking. Third, the surgeon's cockpit has been designed to enable a 3D view with flexible eye position setting [5]. This will contribute to relaxing surgeons' eyes. RARC requires a long operative duration; however, hinotori can decrease a surgeon's fatigue. Without the need to dock the arms, the assistant surgeon can easily access the laparoscopic device. In fact, in our institute, the assistant surgeon can behave freely and comfortably around the patient. Additionally, surgical costs are not significantly different between the two systems.

Although this was our first case of ICNB using hinotori, the perioperative outcome was satisfactory. Sim et al., in their retrospective study involving 73 patients who underwent RARC with ICNB using da Vinci Xi, reported operative time, blood loss, and LOS of 452 min, 347 mL, and 16.5 days, respectively [6]. Our results are comparable with those. In contrast, Hosseini et al., in their prospective study involving 158 patients, reported operative time, blood loss, and LOS of 359 min, 300 mL, and 8 days, respectively [7]. These results are more favorable compared with ours. However, our case represents the first time to conduct RARC with ICNB using hinotori worldwide. Therefore, time reduction and shorter hospitalization might be achieved with experience involving more cases.

Various novel robotic surgical systems have been operated in real‐world clinical settings; for example, RARC with ICNB using Hugo RAS (Medtronic, Minneapolis, MN, USA) was reported by Rocco et al. [8] They indicated that it is a feasible procedure that can reproduce all surgical steps comparable with the existing system.

Several problems arise when conducting RARC using hinotori. There are no sealing or stapling instruments for the system. Therefore, currently, we are using laparoscopic devices alternately. Thus, there is an urgent need to develop suitable instruments for hinotori. Also, since only a small sample size has been reported to date with this setting, further comparative studies will be required involving patients undergoing RARC to determine whether hinotori promotes favorable outcomes compared with existing surgical robot systems. We previously reported RAPN, RARN, and RAA using hinotori [9, 10, 11]. We will endeavor to continuously report on the utility of the hinotori surgical system.

In conclusion, this is the first report on the experience of RARC with ICNB using hinotori from Japan. This surgery could be successfully completed and result in a favorable perioperative outcome. We hope that the results of our surgical experience will highlight the usefulness of hinotori.

## Ethics Statement

The present protocol was approved by the institution's optimally constituted ethics committee (Approval No. 21–090).

## Consent

Written informed consent for releasing this case report and accompanying images has been obtained from the patient.

## Conflicts of Interest

Hideaki Miyake is an Editorial Board member of the International Journal of Urology and a co‐author of this article. To minimize bias, he was excluded from all editorial decision‐making related to the acceptance of this article for publication. All other authors declare no conflicts of interest.
